# Influence of flooding duration and aeration on saplings of ten hardwood floodplain forest species

**DOI:** 10.1371/journal.pone.0234936

**Published:** 2020-06-30

**Authors:** Melanie Schindler, Lisa Jungmann, Tobias W. Donath, Kristin Ludewig

**Affiliations:** 1 Division of Landscape Ecology and Landscape Planning, Research Centre of Biosystems, Land Use and Nutrition (IFZ), Justus-Liebig-University Giessen, Giessen, Germany; 2 Department of Landscape Ecology, Institute for Natural Resource Conservation, Kiel University, Kiel, Germany; 3 Applied Plant Ecology, Institute of Plant Science and Microbiology, Universität Hamburg, Hamburg, Germany; Katholische Universitat Eichstatt-Ingolstadt, GERMANY

## Abstract

Alluvial floodplain forests have been reduced drastically in many parts of Europe, due to deforestation, the transformation to settlement and expansion of agricultural areas. Although they have been heavily modified for centuries, generalized frameworks for their management are scarce and the complex interactions between the physical environment and biological processes are often not fully understood. As the zonation of woody species in floodplains is mainly determined by hydrological conditions, flooding tolerance can be regarded as a key factor for the successful establishment of woody species. Furthermore, the oxygen level of the flooding water might affect the responses to flooding. We examined the influence of flooding duration in combination with oxygen supply by aeration on the foliar injury and growth of six-week-old saplings of ten woody species, under controlled common garden conditions. Six of them are considered to be flooding tolerant whereas four are intolerant. In addition, seven are native whereas three are non-native species. During the experiment, the saplings were exposed to partial flooding of different durations (k = 3; three, six and nine weeks) and oxygen levels (k = 2; aerated and not aerated). For comparison, we included an unflooded control. We recorded foliar injury, plant height, number of leaves and stem diameter. We also included a long-term recovery period. Whereas foliar injury decreased for most species with increasing flooding duration, the typical floodplain forest species, classified as flooding tolerant developed better. The differences in species response to flooding could be most likely explained by their ability to react to the resulting stress in morphological, physiological and metabolic terms irrespective whether they are native or not. In addition, the inclusion of a recovery period seems to be important for the assessment of flooding tolerance.

## Introduction

Riparian zones are the interface between aquatic and terrestrial ecosystems [1]. Due to their small-scale heterogeneity, they are hot spots of species richness and belong to the ecosystems with the highest biodiversity on earth [2,3]. However, due to deforestation, transformation to settlement and expansion of agricultural areas, alluvial floodplain forests have been drastically reduced in Germany and also in many other parts of Europe [4]. The hydrological regime of the majority of floodplains have been dramatically changed because of the highly regulated rivers. The floodplains get more and more disconnected by dikes resulting in strongly altered flooding frequency and flooding duration [4]. The remaining forests are highly fragmented, with the need for afforestation to re-connect fragments and increase connectivity. Although they have been heavily modified for centuries [5,6], generalized frameworks for their management are scarce [7–9]. In addition, the altered dynamics of riparian ecosystems can trigger the establishment and spread of non-native tree species in floodplains [6,9–14]. On top of this, climate change induced impacts on the alluvial plant communities add to the uncertainty about adequate conservation and restoration measures [15,16]. Whilst the recovery of floodplain forests is one of the most important objectives in alluvial restoration and for the conservation of biodiversity [4], the complex interactions between the physical environment and biological processes are not fully understood, which complicates restoration efforts [17].

Under natural conditions, the zonation of woody species in floodplains is mainly determined by the hydrological regime [18]. In these systems, even minor variations in frequency and duration of flooding result in distinct differences in species composition [19]. Generally, there are two main floodplain forest types which can be distinguished [18,20,21]: softwood floodplain forests are found at sites with more frequent and prolonged flooding and hardwood floodplain forests are found at sites flooded less frequently and for shorter time [22].

A characteristic consequence of flooding is the temporary or permanent water saturation of soil pores, which causes substantial stress to terrestrial plants [23]. Thus, the species that grow in such habitats must be adapted to the changing water levels and flooding [24,25]. Flooding tolerance is therefore a key factor for the successful colonization of floodplain forests by plant species [26,27]. Particularly, the duration of flooding appears to strongly determine the survival of plants [18,28]. Generally, the longer trees are exposed to flooding the greater the damage is [26]. The main problem during flooding is the shortage of oxygen due to the slow diffusion rates of gases in water [26]. Thus, a reduction in oxygen availability results in a decreased photosynthesis [26], which in turn leads to a decline in growthrate, loss of biomass and eventually the death of the flooded plants [29]. However, not all species are equally vulnerable to flooding [30–33]. Therefore, flooding tolerant shrub and tree species have developed several adaptations to tolerate or avoid the effects of flooding [34]. Most of the adaptations to flooding are morphological adaptations [26], such as hypertrophied lenticels, aerenchyma tissues and adventitious roots, which increase the uptake of oxygen by aerial tissues and promote oxygen transportation into the root system [35]. However, knowledge about the flooding tolerance of most Central European tree and shrub species is still incomplete [26]. In addition, conclusions on flooding tolerance have been mostly based on the responses during or immediately after the stress period, although it is the sum of plant behaviour both during and after the stress. Having survived flooding, poses new stress factors for plants during recovery [36]. Therefore, plant recovery after flooding has often been overlooked, but seems to be very important to avoid misjudgements [36].

The success of trees in flooded areas also depends on their age. Plants are most vulnerable during the sapling phase [37,38]. In contrast, adult trees are less sensitive to flooding than saplings of the same species [39–42]. Thus, even the species rated as tolerant to flooding in later phases may be quite sensitive during the sapling stage [26].

Besides the stressful physical environment, another threat to the regeneration of the native plant communities in the hardwood floodplain forests is the invasion by non-native tree species due to altered hydrological dynamics [43]. Kawaletz et al. [44], for example, showed that once established, non-native trees effectively reduce the recruitment of native species saplings [45,46]. Consequently, knowing the requirements of non-native species during their early establishment phase can have important implications for the management of the non-native species in alluvial forest [47,48].

In summary, the success of restoration projects is based on the knowledge of the ecological, hydrological and geomorphological processes, as well as the flooding tolerance of the characteristic species based on the assessment after a certain recovery period [26]. While most of the studies on the flooding tolerance of trees have focussed on softwood species, only a limited number of studies included hardwood forest species [49,50], even though they are usually much more threatened by human activities [51]. Consequently, to reduce this gap in knowledge, the effects of flooding on the survival and development of hardwood forest species needs to be investigated in more detail.

Therefore, we investigated the tolerance of six-week-old saplings of ten woody species to flooding in a controlled pot experiment. We included trees and shrubs, flooding tolerant and flooding intolerant species as well as natives and non-natives. In detail, we examined the influence of flooding duration in combination with oxygen supply by aeration on the foliar injury and growth of the saplings under controlled common garden conditions. As response variables, we assessed plant growth in terms of plant height, number of leaves and stem diameter as secondary growth. Specifically, we tested the following hypotheses:

1. With increasing flooding duration, the foliar injury and growth of the investigated woody species will be negatively affected, showing reduced plant height, less leaves and a reduced secondary growth. These negative flooding effects will be more pronounced for the intolerant species compared to the flooding tolerant species irrespective whether they are natives or non-natives.
2. The decrease in foliar injury and growth will be less pronounced with the addition of oxygen by aeration to the flooding basins.

## Methods

### Study species

We used woody species that show different degrees of flooding tolerance (Table 1). As representatives for flooding tolerant hardwood floodplain forest species of northern Central Europe, we selected *Q*. *robur* L., *F*. *excelsior* L., *C*. *sanguinea* L. and *C*. *monogyna* Jacq. We also included flooding intolerant species of northern Central Europe such as *Acer pseudoplatanus* L., *Sambucus nigra* L. and *Sorbus aucuparia* L., which already occur more often at higher altitudes of hardwood floodplains because of the increasing drought. In addition, we used three tree genera: Acer, Fraxinus and Quercus. For each of the three genera we tested a native and a non-native species (Table 1). The non-native species were *Acer negundo* L., *Fraxinus pennsylvanica* Marshall and *Quercus rubra* L. The reason to include non-native species was that in floodplains already non-native species such as *A*. *negundo* and *F*. *pennsylvanica* occur. In order to test the hazard potential of another non-native, but flooding intolerant species, we chose *Q*. *rubra* L. From the total of the above-mentioned species, six were classified as flooding tolerant and four as flooding intolerant (Table 1). We chose a rough classification to differentiate only between flooding tolerant and flooding intolerant species. The nomenclature of plant species follows Jaeger et al. (2017).

**Table 1:** Information about study species, their family, origin and whether they are considered to be flooding tolerant in the literature (including citations).

Species	Family	Origin	Flooding tolerance
***Acer negundo***	Sapindaceae	North America, alluvial forests [52]	Yes [52,53]
***Acer pseudoplatanus***	Sapindaceae	native	No [49,54]
***Cornus sanguinea***	Cornaceae	native	Yes [55]
***Crataegus monogyna***	Rosaceae	native	Yes [34,49]
***Fraxinus excelsior***	Oleaceae	native	Yes [55,56]
***Fraxinus pennsylvanica***	Oleaceae	North America lowlands [57]	Yes [57,58]
***Quercus robur***	Fagaceae	native	yes [55,56,59]
***Quercus rubra***	Fagaceae	Eastern North America [60]	No [61]
***Sambucus nigra***	Adoxaceae	native	No [55,62,63]
***Sorbus aucuparia***	Rosaceae	native	No [53,54]

### Experimental setup

In a pot experiment, we investigated the influence of the flooding duration, in combination with or without oxygen supply by aeration with an aquarium pump, on the growth of the studied saplings. Three factor levels of flooding durations *(k = 3)* were tested: short (three weeks), medium (six weeks) and long (nine weeks). In addition, we applied two oxygen treatments per flooding duration *(k = 2)*, which means that per flooding duration treatment the water in one basin was aerated with 9 l*min^-1^ of air using an aquarium pump (Model AIR-8000) while the other was not. In addition, we had a control group without flooding and oxygen supply. In total, seven treatments were performed. For each species, seven replicates per treatment were conducted. With the ten above-mentioned species, this resulted in a total number of 490 plant individuals.

Seed collection took place along the Middle Elbe River in mid-September 2016 for the species *C*. *sanguinea*, *C*. *monogyna*, *S*. *nigra* and *S*. *aucuparia* and in early October 2016 for the other species (in a range of NW 53° 21’ N, 10° 42’ E and SE 52° 58’ N, 11° 38’ E). The seeds were sampled from trees in the active and fossil floodplain along the Middle Elbe River. The collected seeds were kept cold and dry with sufficient air moisture until they were cold-wet stratified in potting soil in a climate chamber at 4°C following [64] (S1 Table). Subsequently, seeds were grown in a greenhouse during April and May 2017. At the end of May, all emerged seedlings were planted into pots (6 cm x 6 cm x 7.5 cm) filled with a 1:1 mixture of sand and commercial potting soil (Fruhstorfer Erde®, Type P, Industrie-Erdenwerke Archut GmbH, Lauterbach/Germany). The flooding experiment was set up at the research station Linden-Leihgestern of the Justus-Liebig University (Giessen, Germany, 50° 32’ N, 8° 41’ E) from June to September 2017. The seven basins consisted of a wooden frame of 1 m^2^ laid out with a 0.2 mm thick waterproof silage film (with the bright side upwards). The experiment was set up outdoors on a paved area exposed to ambient light, wind, temperature and precipitation. Each treatment group was randomly assigned to one of the seven basins while the pots with the saplings were randomly distributed within the basins. This setup resulted in a split-plot design because each treatment was located in one block (i.e. basin) to ensure manageability of the flooding/oxygen treatments. For the flooding treatment, all basins with the exception of the control basin, were filled with tap water up to 2 cm above the pot rim, i.e. plants were not completely covered by water. If necessary, evaporated water was refilled, so that water levels were kept constant during the flooding period. At the end of the flooding treatment, the water was removed. Subsequently, those plants as well as the plants of the control were irrigated as required.

The experiment started on June 9^th^ 2017. The oxygen content of the water was measured on a weekly basis. During the first two weeks, we used a JBL Testlab (JBL Testlab 25502, JBL, Neuhofen/Germany). Using this test set, the oxygen content was determined by a color change after the addition of two reagents. To increase the accuracy, we used an oximeter (WTW Oxi 325, Xylem Analytics Germany, Weilheim/Germany) to measure the oxygen content from the third week on. The oxgen content in the basins with aeration was significantly higher compared to the basins without aeration (*p*-value = 0.001; S1 Fig). In addition, the oxygen content decreased significantly from short to long flooding duration (*p*-value = 0.034; S1 Fig).

After the ninth week, when all individuals completed the flooding treatment, the plants were carefully repotted into larger pots (11 cm x 11 cm x 12 cm) to ensure optimal conditions. On September 1^st^, after twelve weeks, including a recovery period of at least three weeks for the longest flooding duration, foliar injury and growth of the individuals were recorded. The plant heights were measured as well as the stem diameter as secondary growth at the soil surface using a caliper. In addition, the number of leaves were counted. For the foliar injury of each plant, we defined five injury classes (1: all leaves without damage, 2: all leaves are fully developed but show damage < 50%, 3: leaves not fully developed and > 50% of them damaged, 4: all leaves damaged or dead, but plant is still alive, 5: plant dead). In June 2018, the foliar injury was recorded again after a recovery period of nine months. During the whole time, the plants were watered when needed.

### Statistical analyses

For the comparison of the survival of the species between the flooded and control individuals and between the measurement after twelve weeks and after one year, we applied a two-way ANOVA. We used the factors *species*, *flooded individuals* (flooded, control) and *time* (12 weeks, 1 year) as well as their interactions together with the parameter ‘survival’ as the response variable. We visually checked diagnostic plots to test the preconditions of ANOVAs (e.g. normal distribution, variance homogeneity). For post-hoc testing, we used pairwise t-test with Holm adjusted *p*-values [65].

We analyzed the effect of the treatments and control on the foliar injury of the saplings by conducting Scheirer-Ray-Hare-Tests [66]. As response variables, the parameters ‘foliar injury after twelve weeks’ and ‘foliar injury after one year’ were used. We computed separate Scheirer-Ray-Hare-Tests for each species for both response variables with the factors *flooding duration* (control, short, medium, long) and *aeration* (control, yes, no) and their interaction [65]. For post-hoc testing, we used the Dunn Kruskal-Wallis multiple comparison with Benjamini-Hochberg adjusted *p*-values [65].

To determine whether there were differences in foliar injury between the flooding tolerant and the flooding intolerant species, the Scheirer-Ray-Hare-Test was used with the same response variables (‘foliar injury after 12 weeks’ and ‘foliar injury after 1 year’). However, not all species were tested separately; rather the factor *flooding tolerance* (yes, no) was included to the factors. In order to investigate the comparision of natives and non-natives, only the three tree genera were used. Therefore, the factor *native* (yes, no) was included to the factors. In addition, to compare the foliar injury after 12 weeks and 1 year, the factor *time* (12 weeks, 1 year) was included to the factors and tested separately for each species.

In a next step, we tested the effects of the above-mentioned factors on the growth of the saplings. Therefore, we used the response variables ‘plant height’, ‘number of leaves’ and ‘stem diameter’ and computed separate split-plot-setup ANOVAs for each species. We excluded dead individuals from this analysis to avoid detrimental effects of zero values on ANOVAs. Before analysis, the variables were standardized using a natural logarithmic response ratio (RR) as suggested by Goldberg and Scheiner [67].

$$RR=ln\;(PT/\bar{PC})$$

This standardization of the parameter value of the treated sample (PT) with the mean value of the control ($\bar{\mathrm{PC}}$) for each species allows species comparision. Effects of the treatments on the survived plants were considered significant (i.e. different from the controls) when 95% CI did not overlap with zero in Figs 4–6. Thereafter, ANOVAs for split plot designs were analysed for the factors flooding duration (short, medium, long), aeration (yes, no) and their interaction for every species and response variable (‘RR plant height’, ‘RR number of leaves’ and ‘RR stem diameter’) separately. For the post-hoc pairwise t-tests Holm adjusted p-values were applied [65].

To determine differences of the growth between the flooding tolerant and the flooding intolerant species, the response variables (‘RR plant height’, ‘RR number of leaves’ and ‘RR stem diameter’ as well as the factors (*flooding duration*, *aeration* and their interaction) were used. Here too, not all species were tested separately; rather the factor *flooding tolerance* (yes, no) was included in the analyses as well as the factor *native* (yes, no) in the analysis for differences between native and non-native species.

For the analysis of significant differences in the oxygen content between the basins, we computed a Wilcoxon Signed Rank test because the preconditions for an ANOVA were not fulfilled and the transformation did not result in the necessary assumptions for normal distribution. For post-hoc testing, we used Dunn’s test with Holm adjusted *p*-values [65].

The significance level of all analyses was set up at α = 0.05. All statistical analyses were carried out using the R software environment [68].

## Results

### Survival

**Fig 1:**
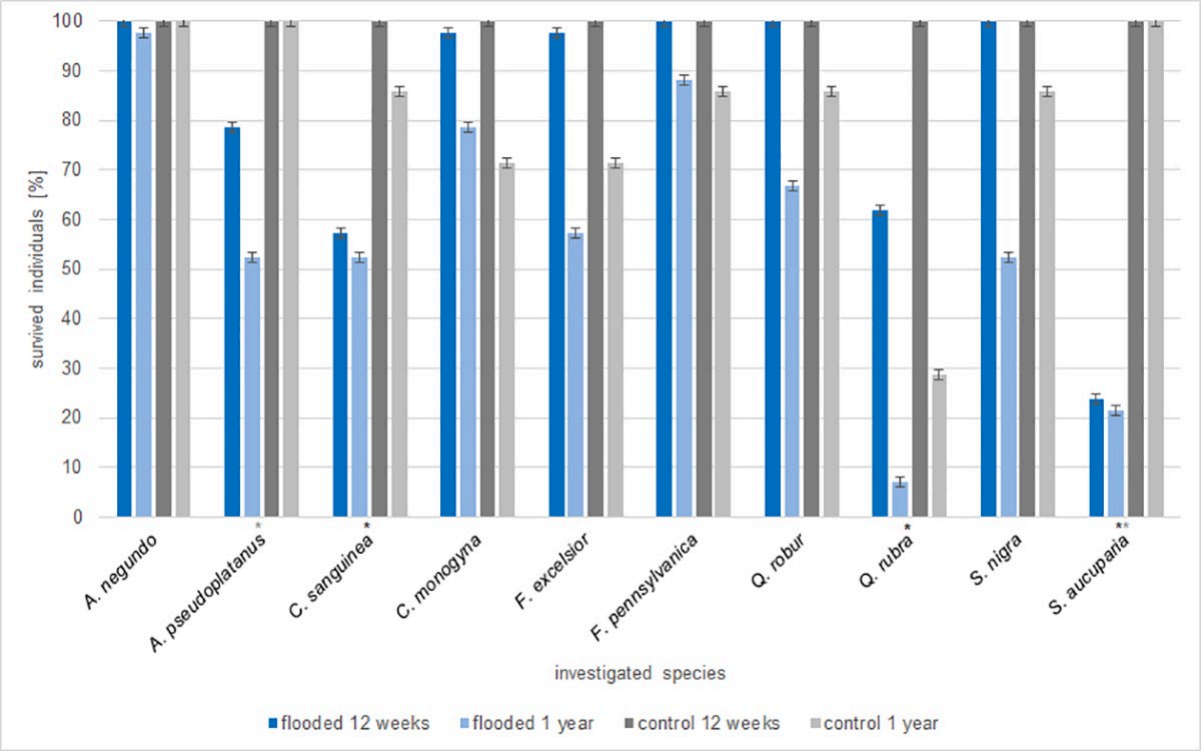
Percentage of the survived individuals of each species in the flooded and control treatment twelve weeks and one year after the experiment started. * indicates significant differences in the survival rate between flooded and unflooded control for that species after twelve weeks; * after one year.

Twelve weeks after the experiment started, 82% of the flooded individuals and 100% of the control survived (Fig 1). With an average survival rate of 92%, almost all individuals of the flooding tolerant species survived the flooding irrespective of the flooding duration and aeration–except for *C*. *sanguinea*, which showed a comparatively low survival rate of only 58%. The flooding intolerant species showed a survival rate of 66% (Fig 1). Significantly more individuals of *C*. *sanguinea* (*p*-value = 0.030), *Q*. *rubra* (*p*-value = 0.048) and *S*. *aucuparia* (*p*-value ≤ 0.001) survived the unflooded control compared to the flooded treatments (Fig 1). One year after the flooding experiment started and after the recovery period of nine months, a further 24% of the flooded individuals were dead (Fig 1). In total, 73% of the flooding tolerant species survived the flooding treatments compared to 33% of the flooding intolerant species. In the control, only 81% of the individuals survived after one year (Fig 1). Especially *Q*. *rubra* showed a low survival rate of only 29% in the control (Fig 1). Significant differences in the survival rate between flooded and control individuals were only visible for the flooding intolerant species *A*. *pseudoplatanus* (*p*-value = 0.017) and *S*. *aucuparia* (*p*-value ≤ 0.001). The survival of the native and non-native species did not differ significantly in relation to the treatments.

In general, the survival rate differed significantly between the species (*p*-value ≤ 0.001) and the time of measurement (*p*-value ≤ 0.001). In the flooded treatments, a significant decrease in the survival rate after one year compared to the measurement after 12 weeks were found for *A*. *pseudoplatanus* (*p*-value = 0.036), *F*. *excelsior* (*p*-value = 0.001), *Q*. *robur* (*p*-value = 0.008), *Q*. *rubra* (*p*-value ≤ 0.001) and *S*. *nigra* (*p*-value ≤ 0.001; Fig 1). For *A*. *negundo* (*p*-value = 0.847), *C*. *sanguinea* (*p*-value = 0.7) and *S*. *aucuparia* (*p*-value = 0.84), almost all individuals that were alive after 12 weeks survived (Fig 1). *Q*. *rubra* (*p*-value = 0.003) is the only species where significantly less individuals even of the control survived one year after the experiment started compared to 12 weeks (Fig 1).

### Foliar injury

**Fig 2:**
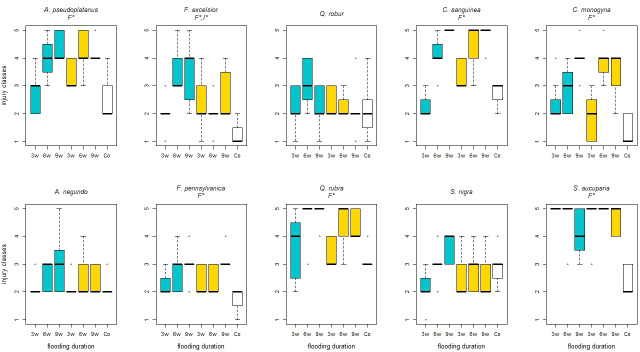
Foliar injury of the species depending on the treatments 12 weeks after the experiment started. Injury classes from 1 (without damage) to 5 (dead); after 3 weeks (3w), 6 weeks (6w) and 9 weeks (9w) flooding duration and unter control (Co) conditions without flooding and aeration.; Blue boxplots: oxygen supply by aeration, yellow boxplots: no aeration; significant differences between the main effects were marked with capital letters (F*–Flooding duration, O*–Oxygen treatment, I*–Interaction between both).

In general, with increasing flooding duration, the foliar injury of most species *A*. *pseudoplatanus* (*p*-value ≤ 0.001), *C*. *sanguinea* (*p*-value ≤ 0.001), *C*. *monogyna* (*p*-value = 0.001), *F*. *pennsylvanica* (*p*-value = 0.009) and *Q*. *rubra* (*p*-value = 0.003) increased twelve weeks after the start of the experiment (Fig 2). Only *A*. *negundo*, *F*. *excelsior*, *Q*. *robur* and *S*. *nigra* showed an almost constant foliar injury over all flooding durations (Fig 2). Significantly less foliar injury of the control compared to the medium and long flooding duration was visible for *A*. *pseudoplatanus* (*p*-values ≤ 0.001), *Q*. *rubra* (*p*-values = 0.002), *C*. *monogyna* (*p*-values ≤ 0.001), *C*. *sanguinea* (*p*-values ≤ 0.001), *F*. *excelsior* (*p*-values = 0.003, 0.001), *F*. *pennsylvanica* (*p*-values ≤ 0.020, 0.001), and *S*. *aucupria* (*p*-values ≤ 0.001). The few surviving individuals of *S*. *aucuparia* showed a very high foliar injury (Fig 2).

The aeration had no significant effect on foliar injury of the species. Whereas the flooding tolerant species showed a significantly lower foliar injury over all flooding treatments compared to the flooding intolerant species (*p*-value ≤ 0.001), the foliar injury of native and non-native species showed no significant difference (*p*-value = 0.096; Fig 2).

**Fig 3:**
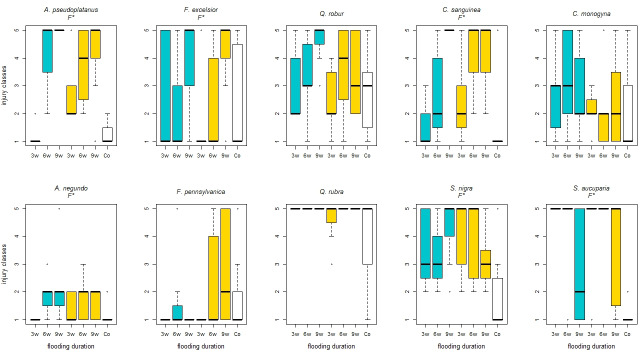
Foliar injury of the species depending on the treatments one year after the start of the experiment, including a nine months recovery period. Injury classes from 1 (without damage) to 5 (dead); after 3 weeks (3w), 6 weeks (6w) and 9 weeks (9w) flooding duration and unter control (Co) conditions without flooding and aeration.; Blue boxplots: oxygen supply by aeration, yellow boxplots: no aeration; significant differences between the main effects were marked with capital letters (F*–Flooding duration, O*–Oxygen treatment, I*–Interaction between both).

One year after the start of the experiment, including a recovery period of nine months, the range of classification of the injury classes increased (Fig 3). There was a significant increase in foliar injury from short to medium flooding duration for *A*. *pseudoplatanus* (*p*-values ≤ 0.001) and *C*. *sanguinea* (*p*-values = 0.042). *S*. *aucuparia* showed a significant increase in foliar injury from medium to long flooding duration (*p*-values = 0.036). The individuals of the control of *S*. *aucuparia* (*p*-values ≤ 0.001) and *S*. *nigra* (*p*-values = 0.03) showed a significantly lower foliar injury compared to the individuals of all flooding durations. The individuals of the control of *A*. *pseudoplatanus* (*p*-values = 0.003, 0.001) and *C*. *sanguinea* (*p*-values = 0.04, 0.001) showed a lower foliar injury compared to the medium and long flooding duration. For all species, the aeration showed no general pattern on the foliar injury (Fig 3).

The flooding tolerant species showed again a significantly lower foliar injury compared to the flooding intolerant species (*p*-value ≤ 0.001). In addition, significant differences between the native and non-native species could be observed (*p*-value = 0.002). Whereas the two non-natives of the genus Acer and Fraxinus showed a significantly lower foliar injury compared to the natives, the reverse was true for the genus Quercus.

A comparison of the foliar injury after twelve weeks and after one year showed different results for the different species (Figs 2 and 3). While the foliar injury of *A*. *negundo* (*p*-value = 0.008), *F*. *pennsylvanica* (*p*-value ≤ 0.001) and *C*. *sanguinea* (*p*-value = 0.03) decreased after one year, the foliar injury of *Q*. *robur* (*p*-value ≤ 0.001), *Q*. *rubra* (*p*-value ≤ 0.001) and *S*. *nigra* (*p*-value = 0.008) further increased (Figs 2 and 3).

### Plant height

**Fig 4:**
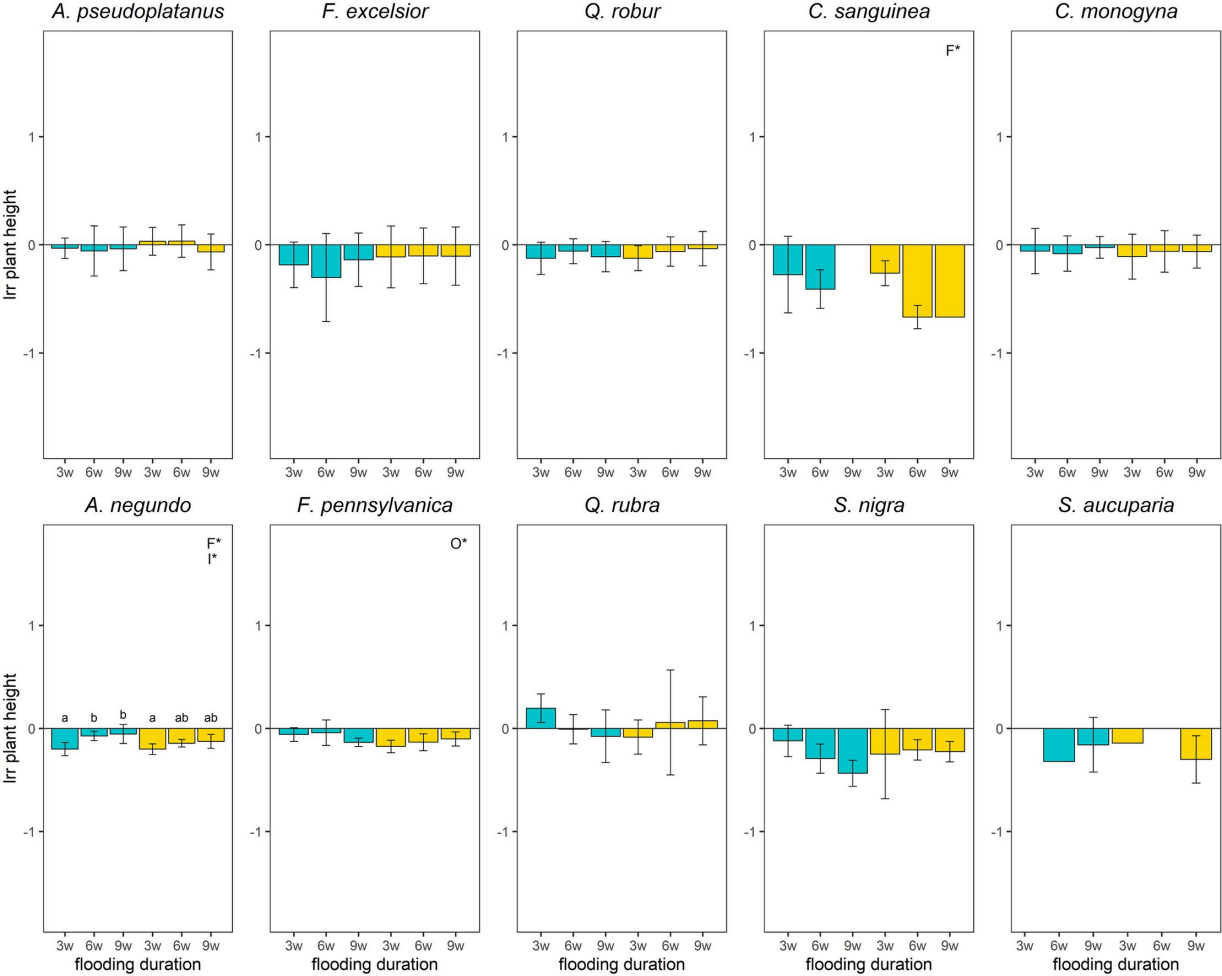
Logarithmic response ratio (lrr) of plant height of the species depending on the treatments 12 weeks after the experiment started. w = weeks, the number prefixed indicates the flooding duration in weeks; Blue boxplots: aeration, yellow boxplots: no aeration; significant differences between the main effects were marked with capital letters in the upper right corner (F*–Flooding duration, O*–Oxygen treatment, I*–Interaction between both); Effects of treatments on survived plants were considered significant (i.e different from the control) when 95% CI did not overlap with zero. Missing bars represent groups with a mortality of 100%. Missing CI represent groups with only one survival individual.

Negative flooding effects on plant height for nearly every treatment compared to the control were significant for *A*. *negundo*, *C*. *sanguinea*, *F*. *pennsylvanica* and *S*. *nigra* (Fig 4). For *A*. *pseudoplatanus*, *F*. *excelsior*, *C*. *monogyna* and *Q*. *rubra*, none of the treatments showed a significant difference in plant height from the control (Fig 4). With increasing flooding duration, the height of *C*. *sanguinea* (*p*-value = 0.03) showed a significantly negative effect, while the significant differences in plant height between the short and medium (*p*-value = 0.002) and between the medium and long flooding duration (*p*-value ≤ 0.001) for *A*. *negundo* showed a less negative effect with increasing flooding duration. Without aeration there was a significantly more negative effect on the plant height of *F*. *pennsylvanica* compared to the individuals with aeration (*p*-value = 0.002; Fig 4).

### Number of leaves

**Fig 5:**
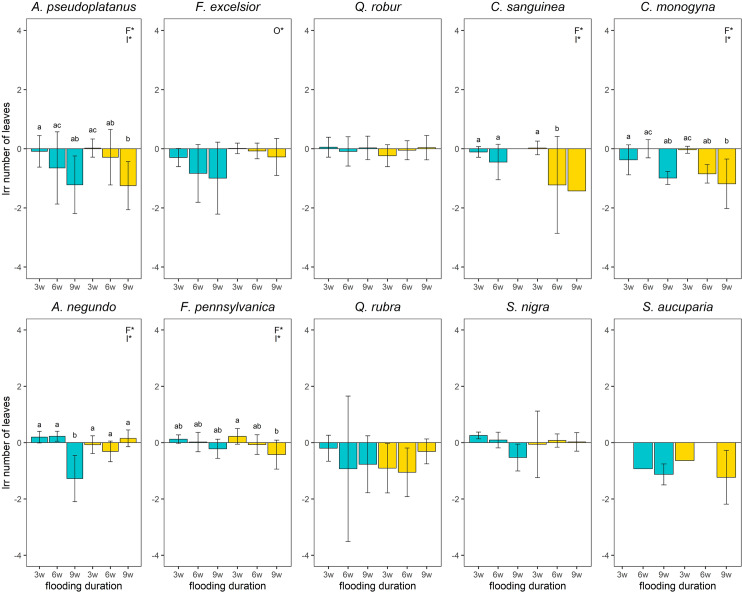
Logarithmic response ratio (lrr) of number of leaves of the species depending on the treatments 12 weeks after the experiment started. w = weeks, the number prefixed indicates the flooding duration in weeks; Blue boxplots: aeration, yellow boxplots: no aeration; significant differences between the main effects were marked with capital letters in the upper right corner (F*–Flooding duration, O*–Oxygen treatment, I*–Interaction between both); Effects of treatments on survived plants were considered significant (i.e different from the control) when 95% CI did not overlap with zero. Missing bars represent groups with a mortality of 100%. Missing CI represent groups with only one survival individual.

The number of leaves of *S*. *aucuparia*, *C*. *monogya* and *A*. *pseudoplatanus* decreased at the long flooding duration irrespective of aeration or not. The combination of long flooding duration with aeration showed also a significantly negative effect compared to the control for *A*. *negundo* and *S*. *nigra* (Fig 5). An increasing flooding duration from short to long showed a significantly negative effect on the number of leaves of *A*. *pseudoplatanus* (*p*-value ≤ 0.001), *A*. *negundo* (*p*-value = 0.04), *C*. *monogyna* (*p*-value ≤ 0.001) and *F*. *pennsylvanica* (*p*-value = 0.003). For *A*. *pseudoplatanus* (*p*-value = 0.03) and *C*. *monogyna* (*p*-value = 0.003), the number of leaves decresed also from medium to long flooding duration and for *C*. *sanguinea* (*p*-value = 0.001) from short to medium flooding duration. No significant difference in the number of leaves compared to the control were visible for *F*. *excelsior*, *F*. *pennsylvanica*, *Q*. *robur* and *C*. *sanguinea*. The oxygen treatment showed only a significantly negative effect on the number of leaves for *F*. *excelsior* with aeration (Fig 5).

### Stem diameter

**Fig 6:**
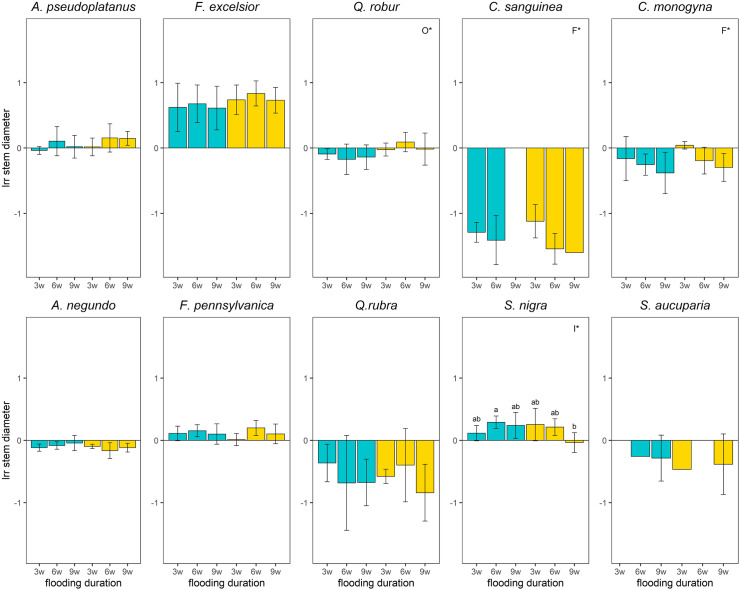
Logarithmic response ratio (lrr) of the stem diameter of the species depending on the treatments 12 weeks after the experiment started. w = weeks, the number prefixed indicates the flooding duration in weeks; Blue boxplots: aeration, yellow boxplots: no aeration; significant differences between the main effects were marked with capital letters in the upper right corner (F*–Flooding duration, O*–Oxygen treatment, I*–Interaction between both); Effects of treatments on survived plants were considered significant (i.e different from the control) when 95% CI did not overlap with zero. Missing bars represent groups with a mortality of 100%. Missing CI represent groups with only one survival individual.

For nearly all treatments, there were significantly negative flooding effects on the stem diameter compared to the control for *C*. *sanguinea*, *A*. *negundo*, *C*. *monogyna* and *Q*. *rubra* (Fig 6). In contrast, the species *F*. *excelsior*, *F*. *pennsylvanica* and *S*. *nigra* showed significantly positiv flooding effects on the stem diameter for most flooding treatments compared to the control. *A*. *pseudoplatanus* and *Q*. *robur* showed no differences in stem diameter (Fig 6). The negative flooding effects increased from short to medium flooding duration for *C*. *sanguinea* (*p*-value = 0.025) and from short to long flooding duration for *C*. *monogyna* (*p*-value = 0.017). The stem diameter of *Q*. *robur* decreased significantly with aeration compared to the treatments without aeration (*p*-value = 0.012; Fig 6).

The flooding tolerant species showed a marginally positive flooding effect on the stem diameter compared to the flooding intolerant species (*p*-value = 0.046). The stem diameter of the native species increased significantly more with flooding than of the non-native species (*p*-value = 0.022; Fig 6).

## Discussion

As expected, the sapling foliar injury of most studied species increased with increasing flooding duration. Furthermore, the flooding tolerant species developed better in terms of survival and foliar injury than the flooding intolerant species after flooding. For example, *A*. *negundo* and *F*. *pennsylvanica*, both classified as highly flooding tolerant in literature [58,69], possess typical adaptations to flooding such as the formation of lenticels and adventitious roots that appear very quickly after five days of flooding [58]. Due to these adaptations, both species showed the best survival and least foliar injury in our study and therefore could pose a risk to native species in alluvial forests. The differences in species response to flooding can be explained mainly by their ability to react to the resulting stress with morphological, physiological and metabolic adaptations, irrespective whether they are native or non-natives [26,70]. This may explain why only a few individuals of *S*. *aucuparia* survived the flooding treatments compared to a 100% survival rate of the control. This species does not develop any of the adaptation structures such as lenticels, aerenchyma or adventitious roots [26]. Our findings were also consistent with the studies of Kozlowski and Brink [53,69], who classified *S*. *aucuparia* as intolerant even to short flooding events. The same reason could explain the lower survival rate of *C*. *sanguinea* compared to *F*. *excelsior* and *Q*. *robur*. *C*. *sanguinea* is only capable of forming adventitious roots, while the other two species can additionally form lenticels and aerenchyma [41,49,54]. Siebel et al. [22] demonstrated that saplings of *C*. *sanguinea* occur only in the higher areas of the hardwood floodplain forest. In addition, not only the formation of these morphological structures is important, but also their quantity [61]. Colin-Belgrand et al. [61] observed that *Q*. *robur* and *Q*. *rubra* developed both, lenticels and adventitious roots, but with a significantly higher intensity for the flooding tolerant and native species *Q*. *robur*. Furthermore, the non-native *Q*. *rubra* is not a typical alluvial forest species and is classified as flooding intolerant [61], an assessment that is–despite its ability to form lenticels and adventious roots–supported by the high mortality rate and foliar injury in the present study. For this reason, *Q*. *rubra* seems to be unable to establish successfully in alluvial floodplain forests and therefore does not pose a risk to native species in alluvial forests. However, we have to take into account that it is difficult to assess invasion impacts because changes in species composition can be slow [71,72] and might take many years before any effects become apparent [73]. On the other hand, it was striking that also many individuals from *Q*. *rubra* died even in the control after one year. Generally, *Q*. *rubra* is not exposed to many abiotic risks except for the risk of late frost. It is more likely that gnawing by rabbits or other rodents caused the mortality. In addition, fungal pathogens for example *Pezicula cinnamonea* or root damage due to parasites like *Gymnopus fusipes* could have led to this high mortality [74].

In general, there are contrary opinions about the flooding tolerance for many species. For the species investigated in this study, the greatest differences in literature were observed for *S*. *nigra*. In our study, all individuals of *S*. *nigra* survived the flooding event, independent of the flooding duration, but showed a high mortality rate and foliar injury after one year. In contrast, the individuals of the control showed a high survival rate and less foliar injury even after one year. In many studies, *S*. *nigra* is described as flooding intolerant [55,62,63]. In contrast, the work of Tremolieres et al. [75], classified *S*. *nigra* as flooding tolerant. Those controversial and contrary assessments show that there are many factors influencing flooding tolerance. Regardless of the abiotic factors such as the “timing”, “depth” and “frequency” of flooding and its biotic factors such as the “developmental stage” of the individuals [26], possibly the biggest problem in most studies is that the classification of the flooding tolerance was based on the observations being made during or immediately following the stress period [36,76]. Most authors of other studies did not include a recovery phase [77]. As we can see in our study, the conclusions drawn may change after a certain recovery period. For example, we also observed a high mortality rate for *A*. *pseudoplatanus*, *F*. *excelsior*, *Q*. *robur* and *Q*. *rubra* after one year. After flooding, plants can face compounding stress factors leading to an increased injury or death [36]. Depending on the growth pattern of the species, flood damage effects may be present up to two years after flooding and therefore recovery time may result in an additional weakening of these species [26]. Another example of the different classifications with regard to flooding tolerance and the importance of the inclusion of a recovery period can be shown for *C*. *monogyna*. Again, there are contrary judgments, because *C*. *monogyna* was partly described as a species with a low flooding tolerance by Glenz [55] while Vreugdenhil et al. [34] described this species as having a higher flooding tolerance than *F*. *excelsior* and *Q*. *robur*. However, the ability of *C*. *monogyna* to cope with flooding is poorly understood, although it has been shown to be able to recover better after flooding, when compared to *F*. *excelsior* and *Q*. *robur* [54]. This is in accordance with our study, which also showed a higher foliar injury for *C*. *monogyna* compared to *Q*. *robur* and *F*. *excelsior* in the long flooding duration after twelve weeks but less foliar injury and better survival rates than both species after the recovery period of nine months. A poor performance during flooding does not necessarily involve a reduced flooding tolerance, as some species can save energy for later recovery [36]. Again, the inclusion of a long-term recovery period seems to be very important for the assessment of flooding tolerance.

When reviewing the growth of the studied species, there were many species, which showed a negative flooding effect on plant height. In literature, the most significant effect found in shrubs and trees affected by flooding is a decline in shoot growth [54,69,78–80]. The species may temporarily suspend their growth during flooding by slowing down their metabolism, thus saving energy and maintaining high carbohydrate reserves [36]. The fact that the flooding intolerant species *A*. *pseudoplatanus* and *Q*. *rubra* showed no differences in plant height compared to the control could indicate that this adaptation strategy is not present in these species. Instead, they show a high mortality rate. In accordance to Frye and Grosse [54], we found no differences in plant height during a partial flooding of up to nine weeks compared to the controls for the flooding tolerant species *F*. *excelsior* and *Q*. *robur*. For those species, growth appears to slow down only when flooding continues for longer periods [23]. For *A*. *negundo*, we could observe a trend towards a greater plant height with increasing flooding duration. In the study by Kozlowski [69], *A*. *negundo* showed a higher plant height compared to the unflooded control. One mechanism for a higher plant height could be an enhanced shoot elongation, which allows the plants to extend their leaves out of the water and thereby remain in contact with the atmosphere [41].

In general, with an increased flooding duration, many species react with a decreased number of leaves, which is in accordance with other references [54]. This reaction could be an adaptive strategy by loosing especially older leaves to save energy, which can be used for survival [29]. On the other hand, leaf loss could also be an indicator that the plants are suffering very badly under flooded conditions. Accross all flooding durations, no changes in the number of leaves were observed for *Q*. *robur*. This is in accordance with the studies of Alaoui-Sossé et al. [23] and Frye and Grosse [54]. The reason could be, as Späth [81] documented, that Quercus saplings were able to sprout new leaves even after being flooded for more than 50 days in the Rhine flood in 1987. No differences in the number of leaves compared to the control were also visible for *F*. *excelsior*, *F*. *pennsylvanica*, *Q*. *robur* and *C*. *sanguinea*. In response to flooding, some species are also able to produce new leaves with a thinner cell wall and cuticle thickness in order to reduce gas diffusion resistance [82]. Another reason for no change in the number of leaves might be that the root system was more affected by the flooding than the leaves.

The flooding tolerant species showed a significantly higher secondary growth compared to the flooding intolerant species. This phenomenon was often observed in flooding tolerant species as they produce more intercellular spaces and lenticels to enhance oxygen transport [41,54,83]. This is also the reason, why there was a significantly higher secondary growth for mostly all treatments compared to the control for *F*. *excelsior* and *F*. *pennsylvanica*. Surprisingly, *A*. *pseudoplatanus* also showed a higher secondary growth for the long flooding duration, which contrasted the study of Frye and Grosse [54] who observed a significantly lower secondary growth after 120 days of flooding for the same species. However, in this study, *A*. *pseudoplatanus* was only flooded up to nine weeks. Analogously, two of the flooding tolerant species, *C*. *monogyna* and *C*. *sanguinea* that are not capable of forming aerenchyma or lenticels showed a significantly lower secondary growth compared to the control, which was also visible for the flooding intolerant species *Q*. *rubra* [54]. These species may have stopped secondary growth to save energy.

Although there are statistical differences between the two oxygen treatments, these rather small differences do not seem to have ecological importance for most of our study species. In general, there was no clear noticeable pattern towards survival, foliar injury and growth. An exception is *Q*. *robur*, which showed a significantly larger secondary growth over all flooding durations without aeration. This may suggest that lenticels were developed even faster in the treatment with a lower oxygen content due to more anaerobic conditions [54]. This would be consistent with the observed enlarged lenticels and thus a higher secondary growth even after three weeks of flooding in the study of Colin-Belgrand et al. [61]. The early formation of morphological structures under more anaerobic conditions could also be responsible for less leave loss of *F*. *excelsior* in the treatment without aeration. The reason for the rather small differences in oxygen content between the oxygen treatments itself, may be that the temperature dependent oxygen solubility in water is resulting in a decreased oxygen content of all basins during summer irrespective of aeration [84]. In addition, the oxygen content may decrease in all basins with increasing flooding duration, because of the activity of microorganisms, which consume oxygen. On the other hand, all basins were set outside, which could also have introduced oxygen even to the basins without aeration due to wind turbulences and the refilled freshwater as it evaporates. Furthermore, the lowest oxygen value of 3.1 mg*l^-1^, was only minimal below the critical oxygen value for fishes and thus possibly not low enough to determine ecological effects on the development of our plants.

## Conclusion

Overall, the typical alluvial floodplain forest species that are classified as flooding tolerant developed better in terms of survival and injury after flooding. This result was also found in the comparison of native and non-native saplings within a genus. Due to the complexity of interacting processes, the knowledge about flooding tolerance of many tree and shrub species is quite sparse and, in some cases, contradictory. However, both flooding tolerant and flooding intolerant species present response patterns that are influenced not only by flooding duration and oxygen content but also by other factors such as seasonal timing, depth and frequency of flooding, as well as the developmental stage of the individuals [26]. Furthermore, plant recovery after flooding seems to be very important to avoid misjudgements in the assessment of flooding tolerance of the species. In order to achieve a better and more comprehensive understanding of the flooding tolerance of woody species, laboratory experiments must be accompanied by field experiments. Nevertheless, experimental studies investigating long term flooding tolerance with regard to flooding duration are urgently required for floodplain forest management.

## Supporting information

S1 Fig Oxygen content of water in mg/l of the flooding basins depending on the treatments. Flooding duration short = 3 weeks, medium = 6 weeks, long = 9 weeks; blue boxplots stand for oxygen supply by aeration, yellow boxplots stand for no aeration; Letters indicate significant differences in oxygen content.
(TIFF)

S1 Table Stratification procedure of the ten study species. (DOCX)

S2 Table Oxygen content [mg/l] of the flooding basins during the test duration. FD = flooding duration: short = 3 weeks, medium = 6 weeks, long = 9 weeks. O_2_ = oxygen supply by aeration; w = week and—means, that no more measurements were done because flooding treatment had finished; * indicate significant differences in oxygen content of the corresponding flooding duration or oxygen treatment.
(DOCX)
