# Supplementary figures and images for: Influence of flooding duration and aeration on saplings of ten hardwood floodplain forest species
Source: PLoS One. 2020 Jun 30;15(6):e0234936. doi: 10.1371/journal.pone.0234936 (PMC7326170; doi:10.1371/journal.pone.0234936)

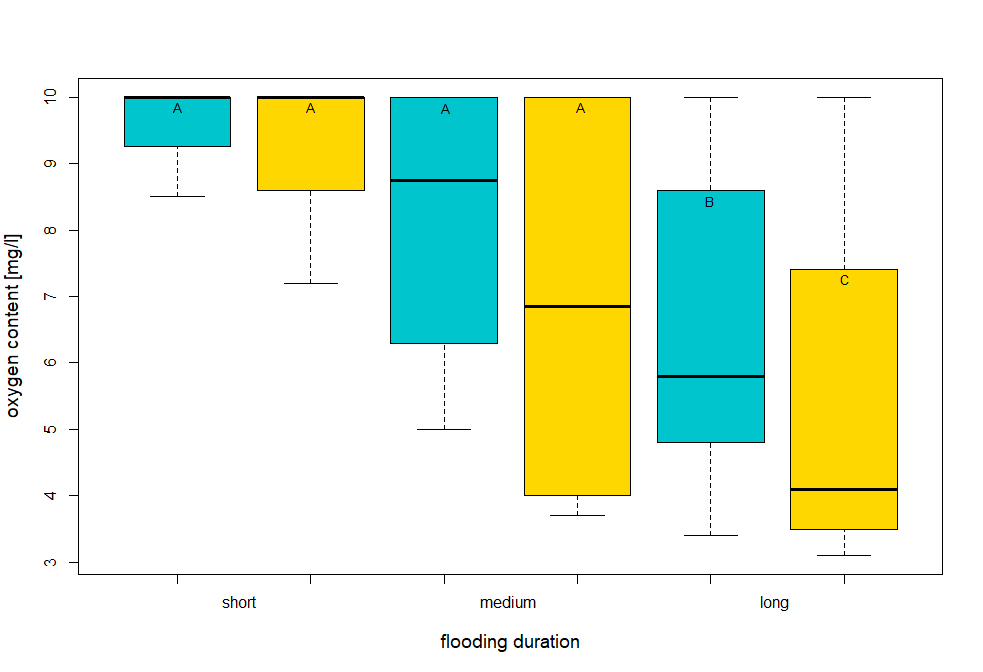

Supplement: S1 Fig — Flooding duration short = 3 weeks, medium = 6 weeks, long = 9 weeks; blue boxplots stand for oxygen supply by aeration, yellow boxplots stand for no aeration; Letters indicate significant differences in oxygen content. (TIFF) [file pone.0234936.s001.tiff]
