# Supplementary material for: Influence of flooding duration and aeration on saplings of ten hardwood floodplain forest species
Source: PLoS One. 2020 Jun 30;15(6):e0234936. doi: 10.1371/journal.pone.0234936 (PMC7326170; doi:10.1371/journal.pone.0234936)
Supplement: S2 Table — FD = flooding duration: short = 3 weeks, medium = 6 weeks, long = 9 weeks. O2 = oxygen supply by aeration; w = week and—means, that no more measurements were done because flooding treatment had finished; * indicate significant differences in oxygen content of the corresponding flooding duration or oxygen treatment. (DOCX) [file pone.0234936.s003.docx]

| **flooding basins** | | | **oxygen content [mg/l]** | | | | | | | | |
| --- | --- | --- | --- | --- | --- | --- | --- | --- | --- | --- | --- |
| Treat-ment | FD | O_2_ | w 1 | w 2 | w 3 | w 4 | w 5 | w 6 | w 7 | w 8 | w 9 |
| 1 | short* | yes | 10 | 10 | 8.5 | - | - | - | - | - | - |
| 2 |  | no | 10 | 10 | 7.2 | - | - | - | - | - | - |
| 3 | medium | yes | 10 | 10 | 8.9 | 8.6 | 6.3 | 5 | - | - | - |
| 4 |  | no | 10 | 10 | 6.9 | 6.8 | 4 | 3.7 | - | - | - |
| 5 | long* | yes* | 10 | 10 | 8.6 | 8.3 | 5.8 | 5 | 4.8 | 3.4 | 4.5 |
| 6 |  | no* | 10 | 10 | 7.4 | 7 | 3.7 | 3.1 | 4.1 | 3.1 | 3.5 |
