# Supplementary material for: Influence of flooding duration and aeration on saplings of ten hardwood floodplain forest species
Source: PLoS One. 2020 Jun 30;15(6):e0234936. doi: 10.1371/journal.pone.0234936 (PMC7326170; doi:10.1371/journal.pone.0234936)
Supplement: Supplementary file 3 [file pone.0234936.s002.docx]

**Year**			2016	2016	2016	2016	2016	2016	2016	2016	2017	2017	2017	2017	2017	2017	2017	2017	2017	2017	2017	2017	2017	2017	2017	2017
**Month**			11	11	11		12	12	12		1	1	1	1		2	2	2		3	3	3		4	4	4
**Week**			45	46	47	48	49	50	51	52	1	2	3	4	5	6	7	8	9	10	11	12	13	14	15	16
**Species**	**soaked**	**heat at 42°C**																								
A. negundo																										
A. pseudoplatanus																										
C. sanguinea	24 h																									
C. monogyna	24 h																									
F. excelsior	24 h																									
F. pennsylvanica	24 h																									
Q. robur		2 h																								
Q. rubra		2 h																								
S. nigra	24 h																									
S. aucuparia	24 h																									

	stored at 25°C
	freezed at -5°C
	stored at 4°C
